# Classification of Targets and Distractors Present in Visual Hemifields Using Time-Frequency Domain EEG Features

**DOI:** 10.1155/2018/9213707

**Published:** 2018-04-01

**Authors:** Sweeti, Deepak Joshi, B. K. Panigrahi, Sneh Anand, Jayasree Santhosh

**Affiliations:** ^1^Centre for Biomedical Engineering, IIT Delhi, New Delhi, India; ^2^Department of Electrical Engineering, IIT Delhi, New Delhi, India; ^3^Department of Computer Engineering & Computer Science, Manipal International University, Putra Nilai, Malaysia

## Abstract

This paper presents a classification system to classify the cognitive load corresponding to targets and distractors present in opposite visual hemifields. The approach includes the study of EEG (electroencephalogram) signal features acquired in a spatial attention task. The process comprises of EEG feature selection based on the feature distribution, followed by the stepwise discriminant analysis- (SDA-) based channel selection. Repeated measure analysis of variance (rANOVA) is applied to test the statistical significance of the selected features. Classifiers are developed and compared using the selected features to classify the target and distractor present in visual hemifields. The results provide a maximum classification accuracy of 87.2% and 86.1% and an average classification accuracy of 76.5 ± 4% and 76.2 ± 5.3% over the thirteen subjects corresponding to the two task conditions. These correlates present a step towards building a feature-based neurofeedback system for visual attention.

## 1. Introduction

In the past decade, motor brain-computer interface (BCI) has witnessed significant progress in the area of rehabilitation. With the promising results in motor BCI, several new directions in this field are emerging. Cognitive BCI (cBCI) is one of them that has drawn the attention of researchers working in the area of BCI. Examples of cognitive signals used for cBCI are the subject's motivation [1], attention orientation, and mental calculation [2]. The fundamental long-term goal of cBCI is to develop therapeutic tools for the treatment of cognitive disorders.

Attention is an important entity for cBCI which works with different sensory inputs, like visual, auditory, and tactile. Cognitive attention offers a person the ability to select the object of interest by ignoring distractors [3]. The information gained with this selection goes to the higher processing mechanism that works like a metacognitive system and results in perception, recognition, and memory formation [4]. The attention paid by a subject to a visual object present at a specific spatial location is called visual spatial attention [5]. A normal human visual system takes about 150 ms to process the visuals [6]. Primarily two known approaches to study visual attention exist: stimulus-driven (bottom-up) and self-driven (top-down) [5] that follow the ventral and dorsal pathways, respectively [7]. The attention capacity of a subject is limited, and the subject can attend two tasks in parallel using its ability of attention shift. It is supposed to work like a spotlight phenomenon and processes only those objects that come under this spotlight [8]. Characteristics of human attention mechanism can be understood from the various theories given in the past like capacity limit theory, attention shift theory, and early and late selection theory [9]. Due to the potential application of selective visual attention in cBCI, it is apparent to explore further the various other aspects of attention.

In the current scenario, researchers are using advanced modalities like fMRI, PET, and fNIRS [5, 10] in cognitive neuroscience research. These techniques are noninvasive and offer a good spatial resolution. However, these techniques suffer lower temporal resolution and higher cost. The present study uses the EEG data to study the selective visual attention due to its good temporal resolution and low cost. The aim of this paper is to develop a classifier that can classify the target and distractor present in opposite visual hemifields using EEG features. These EEG features vary with the task conditions and cognitive load provided. Delta subband provides the best separation between low and high cognitive loads [11]. Previous works suggest that theta band oscillations in the hippocampocortical feedback loop reflect the encoding of new information. Upper alpha oscillations in the thalmo-cortical feedback loop reflect the search and retrieval process in long-term memory [12]. Alpha power is higher for target intake during the attention task while beta band power increases preceding the correct response and does not change in case of the erroneous response [13, 14]. Gamma band power increases in the contralateral hemifield when the subject attends to a stimulus [15, 16]. Temporal evaluation of alpha and beta during spatial visual task indicates a decrease in alpha band power in a time window of 375–500 ms and an increase in beta band power of 500–875 ms [17]. Also, the modulation of EEG rhythms exists during preparatory attention interval. Preparatory attention is associated with the alpha power decrease in the left and right temporal and occipital areas and the beta power decrease in the bilateral occipital, left frontal, and middle frontal occipital [16, 18]. Some other EEG features used for the cognitive load measurement include log variance, Hjorth parameters [19], spectral entropy, spectral edge frequency, intensity weighted mean frequency, and intensity weighted bandwidth [11].

Despite previous significant research in cognition-related studies, it is of utmost importance to extrapolate EEG-based research in attentional orientation for classification purposes in neurofeedback applications. In most of the target versus nontarget classification problems, the task protocols involve the targets and distractors appearing at the central visual field or center of the screen. Such tasks imitate a very different scenario than the real-life situations where target and distractors may appear at different spatial locations or visual fields. The present work involves the classification of the target and distractor present in different visual hemifields while the subject attends the objects using peripheral visual attention. This is more like a real-life situation, for example, looking at the road while attending to or ignoring the objects appearing on both sides of the road. This paper explores the two task conditions: the first task condition compares the activity while the subject attends to the target object present in the left visual hemifield, ignoring the distractor present in the right visual hemifield. The second task condition compares the activity while the subject attends to the target object present in the right visual hemifield, ignoring the distractor present in the left visual hemifield.

In this work, we will explore different EEG features and classifiers are developed further using most relevant features. The results would facilitate the development of a neurofeedback system for selective visual attention [20–22].  Section 2 describes the materials and methods used in the study: task description, data preprocessing method, and methodology followed.  Section 3 gives the details of channel selection, statistical analysis, and classification performed.  Section 4 discusses the results.

## 2. Materials and Methods

### 2.1. Task Description

**Figure 1:**
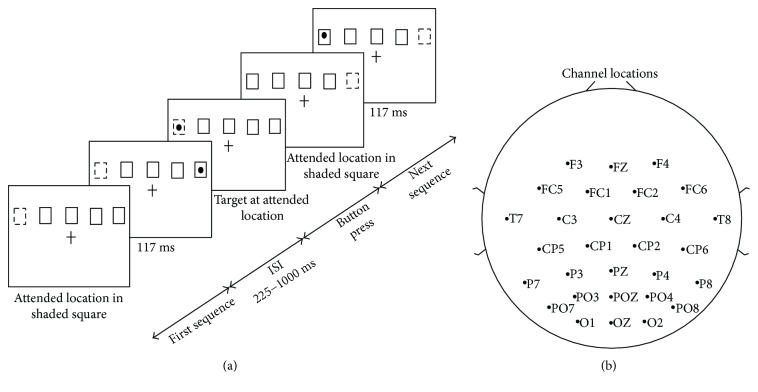
(a) A sequence of the task trial. (b) EEG scalp electrode placement locations.

This study uses the publically available EEG dataset acquired by Jeanne Townsend in the laboratory of Eric Courchesne at UCSD.  Figure 1(a) gives an illustration of one sequence of the task trial presented to the subjects. The task presented five spatial locations represented by square boxes placed from left to right and located 0.8 cm above the central midline of the computer screen along with a center fixation cross present on the computer screen. During each 76-second task block, subjects focused covertly at the attended stimulus location which is presented with a differently shaded box. The attended location is shown in Figure 1(a) by a dashed square box for the illustration purpose. In each task block, 100 disk stimuli appeared randomly at different spatial locations bounded in the square box. Stimuli appeared for a duration of 117 ms at a different spatial location with an interstimulus duration of 225–1000 ms [23]. Subjects responded via a thumb switch, as soon as stimuli appeared at the attended location. The next sequence begins after this. Data was recorded for such thirty task blocks. This paper analyzes the activity with respect to the two conditions where the subject attends to the target present in the left and right visual hemifields while ignoring the distractor present in right and left visual hemifields, respectively.

### 2.2. EEG Recording and Signal Processing

This study uses the publically available EEG dataset acquired by Jeanne Townsend in the laboratory of Eric Courchesne at UCSD. EEG data collected from 13 (two female, eleven male; ages 22–40 years) healthy right-handed subjects performing a visual spatial attention task was recorded from 29 scalp electrode locations using an EEG standard electrode cap (Electro-Cap International Inc.) and two EOG (electrooculogram) electrodes with a sampling frequency of 512 Hz.  Figure 1(b) shows the location of EEG scalp electrodes placed for recording data. The data were collected with reference to the right mastoid electrode position within the analog passband of 0.01–50 Hz. Additional digital filtering was done using 4th-order Butterworth band-pass filter in the range of 0.1–45 Hz, and the signal was average rereferenced. The Automatic Artifact Removal (AAR) v1.3 toolbox based on the blind source separation principle was used to remove the eye blinks and muscle artifacts [24]. EEG analysis involved an epoch length of 800 ms, starting 200 ms before the stimulus onset to 600 ms after the stimulus offset, over 90 trials from each subject.  Figure 1(b) shows the scalp electrode locations.

**Figure 2:**
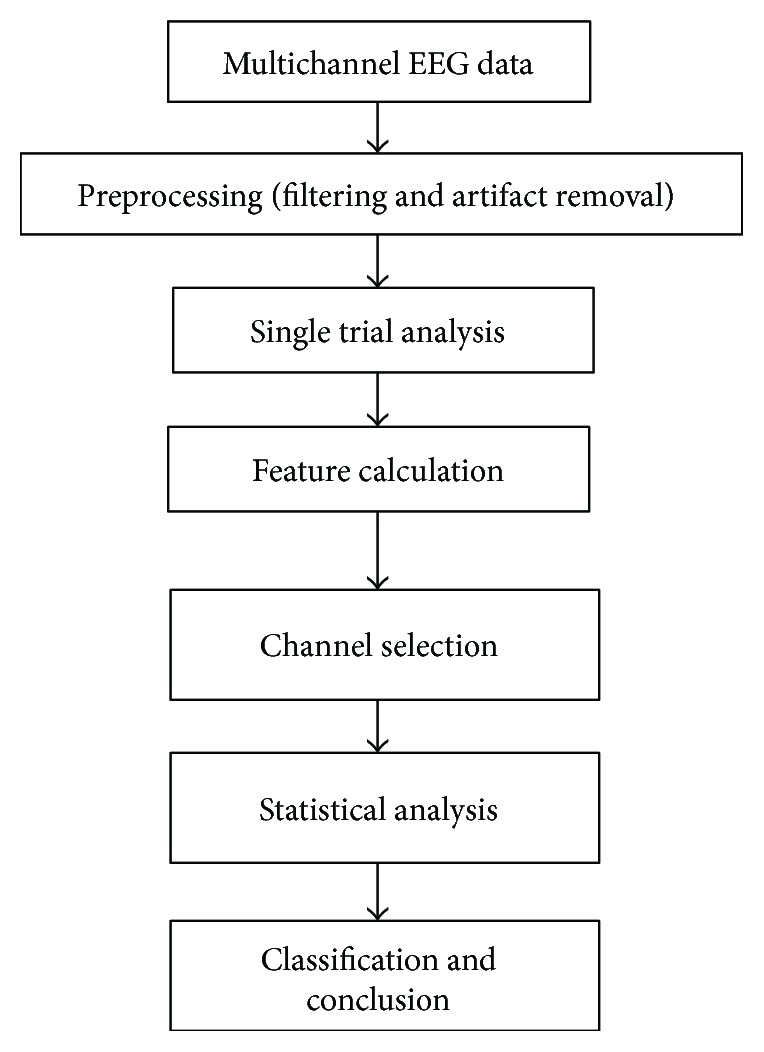
Flowchart for feature-based EEG data analysis.

The selected data is divided into four datasets, namely A, B, C, and D; the summary is given in Table 1. Dataset A corresponds to the activity while the subject attends to the left visual hemifield and the target appears at the same location in the left visual hemifield. Dataset B corresponds to activity while the subject attends to the left visual field and the distractor appears in the right visual hemifield. Dataset C represents the activity while the subject attends to the right visual hemifield and the distractor appears in the left visual field. Dataset D represents the activity while the subject attends to the right visual field and the target appears at the same location in the right visual hemifield. Task condition 1 compares the dataset A and dataset B. The task condition 2 compares the dataset C and dataset D. In other words, the first task condition compares the activity while the subject attends to the target object present in the left visual hemifield ignoring the distractor present in the right visual hemifield. The second task condition compares the activity while the subject attends to the target object present in the right visual hemifield ignoring the distractor present in the left visual hemifield.  Figure 2 demonstrates the flow chart of the complete signal analysis process.

### 2.3. Feature Extraction

EEG features studied are discussed briefly in this section. These features were selected based on their applications in biomedical signal processing as discussed in Introduction. In this work, we are trying to explore the utility of these features in attentional studies:

(1) Hjorth complexity (*H*_complexity_): it is a measure of the spread of the spectrum and represents the change in frequency [25].
  (1) $$\begin{array}{c} H_{complexity}=\frac{std\left(d^{2}X/{dt}^{2}\right)std\left(X\right)}{{\left(std\left(dX/dt\right)\right)}^{2}}, \end{array}$$

where std is standard deviation function.

(2) Hjorth mobility (*H*_mobility_): it is a measure of mean frequency.
  (2) $$\begin{array}{c} H_{mobility}=\left(\frac{std\left(dX/dt\right)}{std\left(X\left(t\right)\right)}\right), \end{array}$$

where std is standard deviation function.

(3) Average frequency: it defines the number of times the signal crosses the zero value.
  (3) $$\begin{array}{c} Average\;frequency=\frac{Total\;zero\;crossing\;points}{Epoch\;duration}. \end{array}$$
(4) Lempel-Ziv complexity (lz-complexity): it is a harmonic variability metric that shows the distinct pattern contained in the sequence as an algorithm scans the data sequence from left to right [25]. To compute the lz-complexity, the signal needs to be decoded first with respect to some threshold value which could be mean or median of the signal. In this way, the signal greater than the threshold which maps to 1 else to 0 to obtain the symbolic sequence is further parsed to obtain the encoded sequence [26]. For an encoded sequence, *s*(*n*) of length *n*, the lz-complexity can be obtained as below:
  (4) $$\begin{array}{c} Lempel-Ziv\;complexity=\frac{s\left(n\right)}{n}. \end{array}$$
(5) Band power: band power can be calculated from the power spectrum of the signal. Power spectrum represents the energy of a signal over the frequency components that it possesses. For a signal *x*, power spectrum can be calculated using
  (5) $$\begin{array}{c} Power\;spectrum=\left(X_{fft}.*conjugate\left(X_{fft}\right)\right), \end{array}$$

where *X*_fft_ represents the Fourier transform of the signal *x*.

(6) Median power frequency (mpf): it is the frequency below which 50% of the total power of the signal is present, calculated over the half of the total area of the power spectrum.
(7) Spectral edge frequency (sef): it is the frequency below which 95% of the total power of the signal is present, calculated over 95% of the total area of the power spectrum.

### 2.4. Channel Selection

Channel selection is an important step as it helps in selecting the channels that can distinguish two or more datasets. This procedure can help in reducing the computational burden by minimizing the number of channels. A number of channel selection methods including filters, wrappers, and embedded methods exist. Among these methods, wrapper methods are good at providing a reliable set of features. The stepwise discriminant analysis (SDA) used in this study is a wrapper method that generates a reliable set of features with multivariate analysis of variance (MANOVA).

A data matrix (*C*_*d*_) is created for each subject corresponding to each EEG feature. The matrix *C*_*d*_ is composed of 29 channels and a total of 180 observations with respect to targets and nontargets for both the task conditions. Rows of the matrix represent the observations, and columns represent the channels as shown:

(6) $$\begin{array}{c} C_{d}=\begin{array}{ccc} a_{i,j} & \ldots & a_{i,j+n}\\ \vdots & \vdots & \vdots \\ a_{i+m,j} & \ldots & a_{i+m,j+n} \end{array}, \end{array}$$

where *i* and *j* represent the rows and column, respectively, *m* represents the number of observations, and *n* represents the number of channels.

The matrix *C*_*d*_ for each EEG feature, for a single subject, is pooled to perform SDA to select the three best performing channels. MANOVA compares the sample means based on the variance-covariance between variables to test the significance difference. MANOVA gives the significant difference value represented by lambda (*λ*). SDA present the channels in descending order of discriminating power *λ*, and the number of channels can further be selected as desired based on the lambda power. In this work, we are selecting three channels.

### 2.5. Statistical Analysis

Repeated measure analysis of variance (rANOVA) was performed to analyze the statistical significance of selected EEG features differentiating the two datasets within the two task conditions. rANOVA investigates the EEG features and task condition interaction over repeated measurements for the two task conditions. It is used to test null hypotheses about the mean. If the mean of the two classes is different, then the null hypothesis rejects. The results of rANOVA are presented in the following form:

*F*(df_fc_, df_error_) = *F* value, *p* = *p* value,

where df_fc_ = degree of freedom of feature and task condition interaction,

df_error =_ degree of freedom of error,

F = critical value,

*p* = significance value.

This test offers Greenhouse–Geisser (pGG), Huynh–Feldt (pHF), and lower bound (pLB) corrections for multiple comparisons to avoid false rejection of the null hypothesis.

### 2.6. Classification and Cross-Validation

In the present work, we need to classify the two classes of the attentional load from targets and distractors. To solve this two-class classification problem, this study compares the three classification approaches, namely, artificial neural network (ANN), *K*-nearest neighbor (KNN), and support vector machine (SVM). The input and target data matrices were constructed for the subject by taking 90 observations from each channel with respect to each dataset. Features were normalized to zero mean and unit variance before classification.

Artificial neural network (ANN): a pattern recognition network architecture with three network layers; namely, the input layer, the hidden layer, and the output layer were used. There were 29 nodes in the input layer, ten nodes in the hidden layer, and one node in the output layer. The tangent sigmoid activation function was used. The network was trained with the Levenberg–Marquardt back propagation method of training using a preset amount of data for the training, testing, and validation. The model was validated using cross-validation methods.

*K*-Nearest neighbor (KNN): the developed KNN model was fitted by means of the Euclidean distance metric between the two nearest neighbors. This model selects the neighbors with a known class from the training dataset and assigns weights to it according to the distance to the space variable using the exhaustive searcher. It made the decision with the majority vote given by selected nearest neighbors. For a space variable *X*, the classifier looks for the nearest neighbor among different classes, say A and B, and assigns the class label having the smallest distance to the space variable *X*.

Support vector machine (SVM): it is a supervised classification model that classifies the data by finding the best hyperplane offering the largest margin between two classes. These hyperplanes are the decision planes that can separate the objects having different class memberships. The developed SVM model mapped the predictor data using radial basis kernel function. Sequential minimal optimization (SMO) approach was used to solve this binary classification problem.

### 2.7. Cross-Validation

The developed classifier models were cross-validated using the *k*-fold cross-validation approach. In this approach, the data is divided into *k* subsamples randomly. At each fold, (*k* − 1) subsamples are used for the training, remaining one for the testing. The process is repeated for each fold, and mean accuracy is calculated from the average of the results of different folds. Also, another cross-validation approach is suggested. This approach works similar to *k*-fold cross-validation except that the criteria for the selection of training and testing datasets is different. Unlike the *k*-fold approach, the datasets are not selected randomly but are selected in a way that there is a maximum time separation in the data points. This cross-validation was performed to avoid the effect of any time-related change in data that may occur during EEG data recording in a block design.

Receiver operating characteristic (ROC) curves were used to compare the performance of the different classifiers. ROC is a plot of the two operating characteristics known as a true positive rate (TPR) and the false positive rate (FPR), where TPR is the probability of detection while FPR gives the probability of false alarm.

## 3. Results

The preprocessed datasets corresponding to the two task conditions were analyzed with the suggested methodology. First, to reduce the system complexity, the channel selection is performed. Feature selection results are given in Table 2. Selected channels are presented in the descending order of their discriminating powers. These results suggest that the parietal and central parietal region electrodes are among the best performing channels to distinguish the targets and distractors.

**Figure 3:**
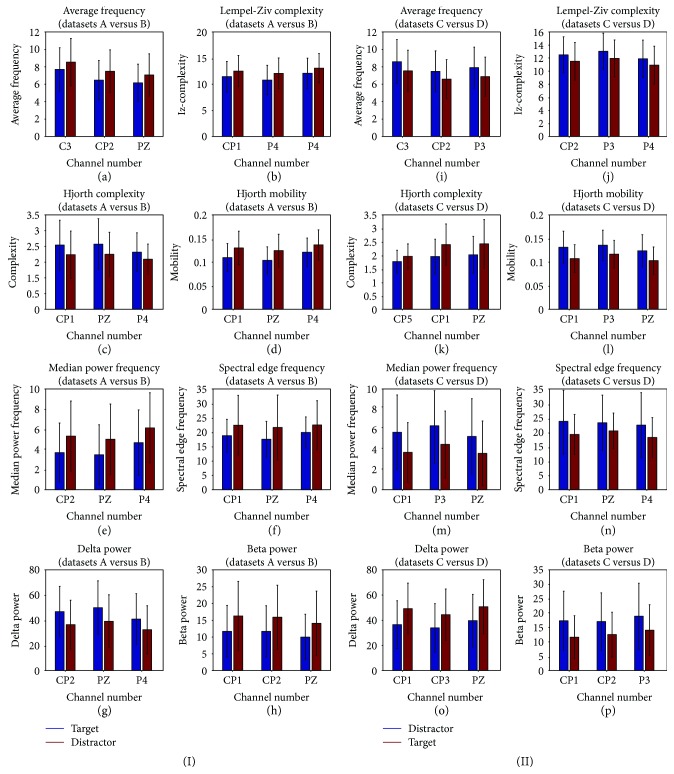
Feature distribution over selected channels for (I) task condition 1 and (II) task condition 2: (a), (i) average frequency; (b), (j) lz-complexity; (c), (k) Hjorth complexity; (d), (l) Hjorth mobility; (e), (m) median power frequency; (f), (n) spectral edge frequency; (g), (o) delta power; (h), (p) beta power.

Further, the distribution of the different EEG features corresponding to the two task conditions with selected channels is studied. This distribution is shown in Figure 3. Figures 3(a) and 3(i) show that the average frequency value is lower for the targets in both task conditions. lz-complexity, which gives the harmonic variability metric, offers greater value for nontargets, suggesting distinct patterns in the signal while ignoring the distractors as shown in Figures 3(b) and 3(j). Hjorth complexity feature's value, which represents the change in frequency, is higher for targets as given in Figures 3(c) and 3(k). It shows that frequency spread is more while attending to targets than nontargets. Figures 3(d) and 3(l) show that Hjorth mobility, which represents the mean frequency, is higher for nontargets. Median power frequency embodying 50% of the total signal power is higher for nontargets as shown in Figures 3(e) and 3(m). Figures 3(f) and 3(n) show that the spectral edge frequency that represents 95% of the total power is also high while ignoring the nontargets. Delta power values are higher for targets for both task conditions as given in Figures 3(g) and 3(o). Figures 3(h) and 3(p) give higher beta power for distractors that advise more activation of the attentional network in inhibiting the nontargets. It can be concluded from Figure 3 that Hjorth complexity and delta power values are higher for targets corresponding to the task conditions 1 and 2 while other features show lower values.

Next, the repeated measure analysis of variance (rANOVA) was performed for all the EEG features extracted from the two task conditions. It was performed using three selected channel features, two datasets, and 90 observations from each dataset. The degree of freedom (DF) was (3 − 1) = 2 for channel features, (3 − 1) ∗ (2 − 1) = 2 for channel feature-condition interaction, and (180 – 2) ∗ (3 − 1) = 356 for error. The rANOVA results show that four among the extracted EEG features exhibit a significant difference for channel feature and condition interaction with a significance level of *p* < 0.1. The result indicates a significant channel feature and task condition interaction with lower bound correction for Hjorth complexity [*F*(2,356) = 6.0145, *p* = 0.0151], Hjorth mobility [*F*(2,356) = 5.62, *p* = 0.0188], delta power [*F*(2,356) = 3.8754, *p* = 0.0505], and beta power [*F*(2,356) = 3.7965, *p* = 0.0529] over the task condition 1. On the other hand, other features including average frequency [*F*(2,356) = 0.42023, *p* = 0.51766], lz-complexity [*F*(2,356) = 0.68079, *p* = 0.41042], mpf [*F*(2,356) = 1.1707, *p* = 0.28073], and sef [*F*(2,356) = 2.3163, *p* = 0.1298] show no statistically significant difference in the target and distractor. For task condition 2, the statistical results display a significant channel feature and task condition interaction with lower bound correction for Hjorth complexity [*F*(2,356) = 3.2531, *p* = 0.0729], Hjorth mobility [*F*(2,356) = 5.0276, *p* = 0.0261], delta power [*F*(2,356) = 3.5058, *p* = 0.0627], and beta power [*F*(2,356) = 2.7321, *p* = 0.0900]. There is no statistically significant channel feature and task condition interaction over the average frequency [*F*(2,356) = 1.012, *p* = 0.31579], lz-complexity [*F*(2,356) = 0.83486, *p* = 0.36211], mpf [*F*(2,356) = 1.937, *p* = 0.16573], and sef [*F*(2,356) = 2.4043, *p* = 0.12278].

The statistical analysis concludes that the Hjorth complexity, Hjorth mobility, delta power, and beta power can significantly differentiate the activity while the subject attends to stationary targets and inhibits the distractors present in another visual hemifield. These selected EEG features are further explored to develop a classifier to classify the target and distractor present in different visual hemifields.

**Figure 4:**
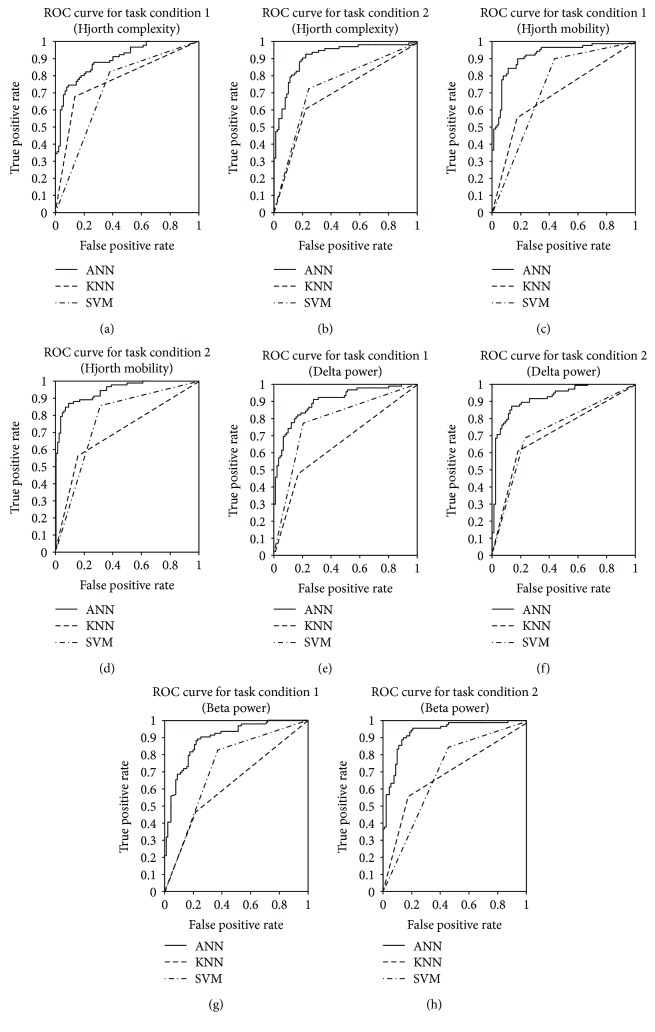
ROC curve for (a) and (b) Hjorth complexity, (c) and (d) Hjorth mobility, (e) and (f) delta power, and (g) and (h) beta power, using artificial neural network (ANN), *K*-nearest neighbor (KNN), and support vector machine (SVM) classifiers during the testing corresponding to task conditions 1 and 2, respectively.

Feature matrices with three selected channel features and 180 observations were prepared for each subject corresponding to all the EEG features for the target and distractor classifications. A comparison of the three different classifiers using the four EEG features for a subject is presented in this section. The ROC curves of Figure 4 illustrate that the artificial neural network (ANN) classifies the target and distractor better than the other two methods. So the details of the classification performance parameters, namely, sensitivity, specificity, and accuracy corresponding to the ANN classifier only, are given in Tables 3 and 4.

Tables 3 and 4 show the mean and maximum values of the classification results obtained from thirteen subjects for the two task conditions using four different EEG features. The classification was performed for each and every subject, and then the mean was taken over the classification results obtained from a population of 13 subjects. These mean values and standard deviation for sensitivity, specificity, and accuracy, corresponding to different features, are given in Tables 3 and 4. These tables also give the maximum value of the classification results obtained.  Table 3 spectates a maximum classification accuracy of 80.6%, 87.2%, 82.2%, and 80% for the task condition 1 using Hjorth complexity, Hjorth mobility, delta power, and beta power, respectively.  Table 4 shows a maximum classification accuracy of 84.4%, 86.1%, 83.3%, and 86.1% for the task condition 2 using Hjorth complexity, Hjorth mobility, delta power, and beta power, respectively.

## 4. Discussion

Selective visual attention is the ability to select the visual information of interest present in the visual field. It is a key to various other skills like perception and recognition and memory as well; it can also affect these skills if there is a problem with it. This paper studies the EEG correlates of visual attention in a spatial attention task. It is important to study features of the EEG as these are very crucial and provide more information than the raw data. These features are the potential candidates that can be used in neurofeedback systems to give feedback about their performance to the subjects. The present study attempts to find the EEG correlates of attention for the task when the subject attends to target objects present in one visual hemifield while ignoring distractor objects present in another visual hemifield. To reduce the system complexity and increase classification accuracy, channel selection is performed [27]. Channel selection performed over EEG features suggest that the channels with the most discriminating power lie in the central-parietal and parietal regions, which are involved in the visual-spatial processing [28]. The rANOVA-based statistical analysis found that amongst the features studied, Hjorth complexity, Hjorth mobility, delta power, and beta power can significantly differentiate the datasets corresponding to the two task conditions. This selection suggests higher beta and lower delta for nontargets, representing higher cognitive demand or working memory load for inhibition which agrees with earlier studies involving targets and nontargets [29]. Hjorth features have been used earlier for cognitive load measurement [19]. The present study explores these features and shows higher Hjorth mobility and lower Hjorth complexity for nontargets which correspond to the mean frequency and change in frequency, respectively.

The classification system is further developed using selected features to distinguish the activities corresponding to targets and nontargets. The importance of such classification lies in applications like cognitive brain-computer interface or neurofeedback system for training where the cognitive control measures are used to control the BCI and train the subjects. Classification accuracy in cBCI is limited by various internal and external factors, like sensory and cognitive, comparative to reasonable accuracy in motor BCI [30, 31]. Due to this limitation, accuracy reported in previous research was restricted to only 75% and 79% in spatial attention tasks [32, 33] using noninvasive techniques. We could reach a maximum accuracy of 87.2% and 86.1% and a mean accuracy of 76.5% and 76.2% over thirteen subjects for the two task conditions, respectively, by using the EEG-based noninvasive method. In this way, a classifier is developed that can classify the peripheral attention paid to targets and distractors present in different visual hemifields. Such a classifier can facilitate the development of an EEG feature-based neurofeedback system for attention [34].

## 5. Conclusion

The present study explores the EEG features that can distinguish the targets and nontargets present in the different visual hemifields. A classification system to classify the targets and distractors present in opposite visual hemifields is proposed in this paper. The analysis is done to optimize the performance of the system. Results provide EEG correlates of selective visual attention that can classify the activities while attending targets and ignoring distractors. Other EEG features can be explored to further increase the classification accuracy and make the possibility of feature-based neurofeedback system feasible.

## Figures and Tables

**Table 1:** Summary of four datasets selected for two task conditions.

Task condition	Task condition 1		Task condition 2	
	Dataset A	Dataset B	Dataset C	Dataset D
Patient state	Attending target object in left hemifield	Ignoring distractor objects in right hemifield	Ignoring distractor objects in left hemifield	Attending target object in right hemifield
Eye fixation	Center	Center	Center	Center
Electrode type	Surface	Surface	Surface	Surface
Electrode placement	International 10-20 placement system	International 10-20 placement system	International 10-20 placement system	International 10-20 placement system
Number of subjects	13	13	13	13
Number of electrodes	31	31	31	31
Number of trials from each subject	90	90	90	90
Epoch duration	800 ms	800 ms	800 ms	800 ms

**Table 2:** Selected channels corresponding to the eight EEG features.

Feature	Task condition 1	Task condition 2
	Selected channels (using SDA method)	Selected channels (using SDA method)
Average frequency	C3, CP2, PZ	C3, CP2, P3
lz-complexity	CP1, PZ, P4	CP2, P3, P4
Complexity	CP1, PZ, P4	CP5, CP1, PZ
Mobility	CP1, PZ, P4	CP1, P3, PZ
Median power frequency	CP2, PZ, P4	CP1, P3, PZ
Spectral edge frequency	CP1, PZ, P4	CP1, PZ, P4
Delta power	CP2, PZ, P4	CP1, P3, PZ
Beta power	CP1, CP2, PZ	CP1, CP2, P3

**Table 3:** Classification results displaying % sensitivity (SN), % specificity (SP), and % accuracy (AC) obtained using ANN for task condition 1 during testing.

Features	Task condition 1					
	Mean			Maximum		
	Sensitivity	Specificity	Accuracy	Sensitivity	Specificity	Accuracy
Hjorth complexity	77.1 ± 7.37	73.6 ± 3.55	75.3 ± 3.72	87.8	78.9	80.6
Hjorth mobility	76.4 ± 5.13	76.6 ± 4.80	76.5 ± 4	88.9	85.6	87.2
Delta power	74.1 ± 5.67	73.0 ± 4.97	73.6 ± 3.93	86.7	80	82.2
Beta power	73.4 ± 5.24	75.8 ± 5.40	74.9 ± 2.85	84.4	83.3	80

**Table 4:** Classification results displaying % sensitivity (SN), % specificity (SP), and % accuracy (AC) obtained using ANN for task condition 2 during testing.

Features	Task condition 2					
	Mean			Maximum		
	Sensitivity	Specificity	Accuracy	Sensitivity	Specificity	Accuracy
Hjorth complexity	73.5 ± 5.12	77.5 ± 6.65	75.5 ± 4.24	81.1	87.8	84.4
Hjorth mobility	77.5 ± 5.36	74.9 ± 6.41	76.2 ± 5.30	86.7	87.8	86.1
Delta power	72.7 ± 5.44	76.0 ± 5.55	74.3 ± 4.76	82.2	85.6	83.3
Beta power	78.6 ± 7.65	72.4 ± 6.38	75.5 ± 5.22	88.9	83.3	86.1

## Data Availability

Data supporting this research article are available from the online Archive of Swartz Center, University of California in San Diego (UCSD), available from http://headit.ucsd.edu/studies/2747adbc-385a-11e3-b29d-0050563f2612.

## Supplementary Materials

Supplementary Materials This manuscript includes a supplementary result file, which contains the classification results obtained.
