# Supplementary material for: Classification of Targets and Distractors Present in Visual Hemifields Using Time-Frequency Domain EEG Features
Source: J Healthc Eng. 2018 Apr 1;2018:9213707. doi: 10.1155/2018/9213707 (PMC5902061; doi:10.1155/2018/9213707)
Supplement: Supplementary Materials — This manuscript includes a supplementary result file, which contains the classification results obtained. [file 9213707.f1.docx]

| **Classification results (%) using ANN over 13 subjects with selected features** | | | | | | | | | | | | | | | | | | | | | | | | |
| --- | --- | --- | --- | --- | --- | --- | --- | --- | --- | --- | --- | --- | --- | --- | --- | --- | --- | --- | --- | --- | --- | --- | --- | --- |
| **Features -** | **Hjorth Complexity** | | | | | | **Hjorth Mobility** | | | | | | **Delta Power** | | | | | | **Beta Power** | | | | | |
|  | **Task Condition** | | | | | | **Task Condition** | | | | | | **Task Condition** | | | | | | **Task Condition** | | | | | |
|  | **1** | | | **2** | | | **1** | | | **2** | | | **1** | | | **2** | | | **1** | | | **2** | | |
| **Subjects** | SN | SP | AC | SN | SP | AC | SN | SP | AC | SN | SP | AC | SN | SP | AC | SN | SP | AC | SN | SP | AC | SN | SP | AC |
| 1 | 85.6 | 68.9 | 77.2 | 81.1 | 73.3 | 77.2 | 84.4 | 72.2 | 78.3 | 76.7 | 73.3 | 75 | 76.7 | 74.4 | 75.6 | 70 | 74.4 | 72.2 | 77.8 | 74.4 | 76.1 | 78.9 | 72.2 | 75.6 |
| 2 | 62.2 | 76.7 | 69.4 | 63.3 | 80 | 71.7 | 77.8 | 72.2 | 75 | 71.1 | 68.9 | 70 | 66.7 | 75.6 | 71.1 | 65.6 | 72.2 | 68.9 | 80 | 61.1 | 70.6 | 80 | 61.1 | 70.6 |
| 3 | 72.2 | 73.3 | 72.8 | 66.7 | 82.2 | 74.4 | 71.1 | 75.6 | 73.3 | 76.7 | 68.9 | 72.8 | 74.4 | 75.6 | 75 | 73.3 | 70 | 71.7 | 65.6 | 76.7 | 71.1 | 77.8 | 67.8 | 72.8 |
| 4 | 75.6 | 67.8 | 71.7 | 71.1 | 74.4 | 72.8 | 75.6 | 73.3 | 74.4 | 68.9 | 76.7 | 72.8 | 74.4 | 66.7 | 70.6 | 71.1 | 68.9 | 70 | 75.6 | 73.3 | 74.4 | 82.2 | 65.6 | 73.9 |
| 5 | 75.6 | 78.9 | 77.2 | 76.7 | 87.8 | 82.2 | 77.8 | 84.4 | 81.1 | 86.7 | 80 | 83.3 | 71.1 | 74.4 | 72.8 | 82.2 | 84.4 | 83.3 | 68.9 | 73.3 | 76.1 | 88.9 | 78.9 | 83.9 |
| 6 | 85.6 | 72.2 | 78.9 | 76.7 | 80 | 78.3 | 74.4 | 78.9 | 76.7 | 82.2 | 73.3 | 77.8 | 74.4 | 67.8 | 71.1 | 78.9 | 76.7 | 77.8 | 73.3 | 83.3 | 78.3 | 84.4 | 65.6 | 75 |
| 7 | 74.4 | 75.6 | 75 | 75.6 | 72.2 | 73.9 | 75.6 | 74.4 | 75 | 78.9 | 73.3 | 76.1 | 72.2 | 77.8 | 75 | 71.1 | 74.4 | 72.8 | 67.8 | 77.8 | 72.8 | 78.9 | 70 | 74.4 |
| 8 | 80 | 68.9 | 74.4 | 71.1 | 70 | 70.6 | 71.1 | 75.6 | 73.3 | 73.3 | 68.9 | 71.1 | 78.9 | 62.2 | 70.6 | 69.4 | 76.7 | 71.6 | 70 | 80 | 75 | 64.4 | 76.7 | 70.6 |
| 9 | 70 | 74.4 | 72.2 | 71.1 | 75.6 | 73.3 | 73.3 | 72.2 | 72.8 | 75.6 | 72.2 | 73.9 | 68.9 | 72.2 | 70.6 | 72.2 | 80 | 76.1 | 70 | 76.7 | 73.3 | 71.1 | 73.3 | 72.2 |
| 10 | 83.3 | 77.8 | 80.6 | 72.2 | 84.4 | 78.3 | 73.3 | 83.3 | 78.3 | 84.4 | 87.8 | 86.1 | 81.1 | 80 | 80.6 | 77.8 | 78.9 | 78.3 | 75.6 | 82.2 | 78.9 | 85.6 | 80 | 82.8 |
| 11 | 87.8 | 76.7 | 82.2 | 81.1 | 87.8 | 84.4 | 88.9 | 85.6 | 87.2 | 83.3 | 86.7 | 85 | 86.7 | 77.8 | 82.2 | 78.9 | 85.6 | 82.2 | 84.4 | 75.6 | 80 | 88.9 | 83.3 | 86.1 |
| 12 | 71.1 | 74.4 | 72.8 | 75.6 | 67.8 | 71.7 | 73.3 | 74.4 | 73.9 | 73.3 | 75.6 | 74.4 | 68.9 | 73.3 | 71.1 | 63.3 | 78.9 | 71.1 | 72.2 | 75.6 | 73.9 | 68.9 | 74.4 | 71.7 |
| 13 | 78.9 | 72.2 | 75.6 | 74.4 | 72.2 | 73.3 | 77.8 | 74.4 | 76.1 | 76.7 | 68.9 | 72.8 | 68.9 | 72.2 | 70.6 | 72.2 | 67.8 | 70 | 73.3 | 75.6 | 74.4 | 72.2 | 73.3 | 72.8 |
| **Mean** | 77.1 | 73.6 | 75.3 | 73.5 | 77.5 | 75.5 | 76.4 | 76.6 | 76.5 | 77.5 | 74.9 | 76.2 | 74.1 | 73.0 | 73.6 | 72.7 | 76.0 | 74.3 | 73.4 | 75.8 | 74.9 | 78.6 | 72.4 | 75.5 |
| **Std.** | 7.37 | 3.55 | 3.72 | 5.12 | 6.65 | 4.24 | 5.13 | 4.80 | 4.00 | 5.36 | 6.41 | 5.30 | 5.67 | 4.97 | 3.93 | 5.44 | 5.55 | 4.76 | 5.24 | 5.40 | 2.85 | 7.65 | 6.38 | 5.22 |
